# Effects of Red Vinasse on Physicochemical Qualities of Blue Round Scad (*Decapterus maruadsi*) During Storage, and Shelf Life Prediction

**DOI:** 10.3390/foods13223654

**Published:** 2024-11-17

**Authors:** Shan Xue, Shuyi Chen, Bohu Liu, Jia Liu

**Affiliations:** 1 College of Biological Science and Technology, Minnan Normal University, Zhangzhou 363000, China; 2 Research Institute of Zhangzhou & Taiwan Leisure Food and Tea Beverage, Zhangzhou 363000, China; 3 Zhangzhou Food Science Research Institute, Zhangzhou 363000, China; 4 Guizhou Academy of Agricultural Sciences, Guiyang 550006, China

**Keywords:** red vinasse, blue round scad (*Decapterus maruadsi*), storage, physicochemical quality, shelf life

## Abstract

A fish processed with red vinasse is a type of Fujian cuisine with regional characteristics. In order to monitor the effect of red vinasse on storage quality and shelf life of blue round scad (*Decapterus maruadsi*) during storage, the changes in fat content, thiobarbituric acid reactive substances (TBARS), composition of polyunsaturated fatty acids (PUFAs), pH value, texture, and sensory quality were studied at different storage temperatures (4 °C, 25 °C, and 37 °C). By analyzing the correlation between changes in sensory qualities and physical and chemical indexes, a first-order kinetic model and the Arrhenius equation were used to build a shelf-life prediction model for blue round scad during storage. The results showed that processing with red vinasse can significantly reduce the malondialdehyde (MDA) production and the decrease in PUFAs, eicosapentaenoic acid (EPA) and docosahexaenoic acid (DHA) (*p* < 0.05) during storage. Based on partial least squares regression (PLSR), the storage temperature and time have a significant impact on the PUFA composition in blue round scad, which changed less when the samples were stored at 4 °C and 25 °C, and they had better nutritional composition of fatty acids at lower temperatures. Among the PUFAs, DHA (C22:6n-3) and EPA (C20:5n-3) had higher relative contents and significantly decreased during storage (*p* < 0.05). Additionally, the processing with red vinasse can slow down the increase in total volatile basic nitrogen (TVB-N) value and pH of blue round scad, maintain the appropriate hardness, elasticity, cohesion and chewability, and improve the overall sensory quality of the fish. In addition, according to the results of model prediction based on TBARS value, the storage shelf life of blue round scad with red vinasse added was 55 d, 2.7 d and 28 h at 4 °C, 25 °C and 37 °C, respectively. The accuracy of the forecast model was high, and the relative errors of the measured values and predicted values were less than 10%. Thus, it not only provided a theoretical basis for the processing and application of red vinasse to Chinese traditional food, but also provided innovative ideas for the safe storage and high-value utilization of blue round scad.

## 1. Introduction

The fish processed with red vinasse is a special food in Fujian, China. Red vinasse, a by-product of *monascus* fermentation of Hongqu rice wine, contains not only nutrients such as protein, crude fat and carbohydrates, but also many antioxidant and antibacterial functional components [1,2]. Studies have shown that monosaccharides produce many functional components in the fermentation process, such as monacolin, gamma-aminobutyric acid, lovastatin, ergosterol, etc., which have liver protection, anti-cancer, anti-oxidation, anti-inflammation, anti-obesity and anti-diabetes properties [3,4]. At the same time, *monascus* and its metabolites can protect hair color and have antibacterial, anticorrosive, antioxidant, and other properties, and have been widely used in many kinds of food such as pastries and beverages [5,6,7,8]. Red vinasse is not only a traditional food originating in China for both medicinal and food purposes, but also a famous food ingredient of Fujian cuisine [9]. Local residents often combine red vinasse with pork or fish to make “red vinasse meat” (meat processed by red vinasse) and “red vinasse fish” (fish processed by red vinasse), which not only has rich and unique flavor, but also can improve the storability of products [9,10].

Blue round scad (*Decapterus maruadsi*), commonly known as balang fish, is a kind of low-value fish distributed around the world. It is the second largest marine fishery product in our country, second only to bandfish, and also an important economic fish off the southeast coast, especially in the Fujian area [11]. It is reported that the blue round scad tastes delicious and has high nutritional value, which is rich in polyunsaturated fatty acids (PUFAs), especially eicosapentaenoic acid (EPA) and docosahexaenoic acid (DHA) [12]. However, blue round scad has some disadvantages, such as soft meat, high red meat content, low pH, insufficient protein elasticity, poor gel properties, and easy spoilage after leaving water. In addition, there are limitations in traditional processing methods, most of which involve fresh fish on ice or dried fish sold in bulk after simple packaging in cartons/plastic boxes. The processing level and high-value utilization degree for this kind of fish are not mature [6,13]. Based on the above analysis, this paper selected a regionally representative red vinasse made in Yuxi county in Fujian province as a raw material, studied the effects of red vinasse on the physical and chemical quality of blue round scad during storage under different temperatures, and built a prediction model of shelf life, in order to provide a theoretical basis for the research and development of local characteristic red vinasse foods and quality monitoring of low-value fish processed with red vinasse.

## 2. Materials and Methods

### 2.1. Preparation of Blue Round Scad Processed with Red Vinasse

Blue round scad (purchased from RT-Mart, Zhangzhou, Fujian, China) processed with red vinasse (produced in Youxi County, Sanming City, Fujian Province, China) was prepared with a method commonly used by local residents to marinate fish. Firstly, blue round scads were washed after removing the scales. The heads, internal organs and bones were removed. Then, the remaining parts were cut into 1.5–2.0 mm fillets with equal thickness, and the excess water on the their surfaces was removed. Secondly, red vinasse was applied in a ratio of 1:1.8 (g/g) all over the fillets, which were then placed in a sterile tray, sealed with plastic wrap, and stored in the refrigerator at 4 °C for 12 h. After marinating, the excess red vinasse was removed, the fillets were wiped clean, and the excess water on their surfaces was removed, thus yielding finished samples of blue round scad processed with red vinasse.

### 2.2. Storage Experiment for Blue Round Scad Processed with Red Vinasse

Samples of blue round scad processed with red vinasse were used as the experimental group, and samples of blue round scad without red vinasse were used as the control group. The fish in the two groups were stored at 4 °C for 0, 3, 6 and 9 days, at 25 °C for 0, 12, 24, 36, 48 h, and at 37 °C for 0, 1, 2, 3, 4, 5, 6 h, respectively (the storage conditions of 4, 25, and 37 °C represented the low-temperature refrigeration, room-temperature storage and accelerated experiments, respectively). The physicochemical indexes of the experimental group and the control group were determined at each time point. All the chemical reagents were purchased from Xilong Science Co., LTD., Shantou City, Guangdong Province, China.

### 2.3. Determination of Physicochemical Indexes

#### 2.3.1. Determination of Fat Content

The fat extraction procedure referred to the method in Xue et al. [14]. Ten grams of blue round scad was minced and placed in a conical bottle, and 120 mL chloroform methanol solution with a volume ratio of 2:1 (*v*/*v*) was added. After fully shaking, the sample was bathed in a constant-temperature water bath at 45 °C for 2.5 h, then filtered. After filtration was completed, 30 mL saturated NaCl solution was added into the filtrate, and the liquid was fully shaken and transferred to a liquid separation funnel for static stratification. The lower layer of fat extract was collected in the separator funnel, filtered and dried with anhydrous Na_2_SO_4_, and concentrated in a 45 °C water bath with a rotary evaporator. Finally, the fat sample was obtained.

#### 2.3.2. Determination of Fatty Acid Composition

The composition of fatty acids was determined by a 7890 B gas chromatographic instrument with flame ionization detector and SH-RtTM-2560 Column (Column 100 m, 0.25 mm ID, 0.20 μm film thickness) with a shutter ratio of 10:1 (*v*/*v*). The sample size was 1 µL, and the carrier gas was nitrogen. The flow rate was 20 mL/min, the inlet temperature was 250 °C, and the detector temperature was 260 °C. The heating procedure for the column box was as follows: the initial temperature was 140 °C, kept for 1 min, then increasing to 240 °C at 4 °C/min and maintaining for 19 min. The retention time of 37 mixed fatty acid methyl ester standards was compared for qualitative analysis of fatty acids, and area normalization was used for quantitative analysis. The polyunsaturated fatty acid (PUFA), saturated fatty acid (SFA), and monounsaturated fatty acid (MUFA) content was expressed as the relative proportion (%) of individual fatty acids to total fatty acids [14].

#### 2.3.3. Determination of Thiobarbituric Acid Reactive Substances (TBARS)

Samples of blue round scad processed with red vinasse were weighed, crushed, and placed in a triangular bottle with 50 mL distilled water and 50 mL 10% trichloroacetic acid solution, then filtered at 27 °C after constant-temperature shaking for 1 h, and the filtrate was used. To 8 mL of the filtrate supernatant, 2 mL 0.06 mol/L thiobarbituric acid (TBA) solution was added. The mixture was shaken well and placed in a constant-temperature water bath at 80 °C for 2 h, then removed and cooled for 30 min to room temperature. Colorimetric measurements were performed at 532 nm, and the absorbance was recorded [15]. Each group of samples was measured in parallel 3 times, and the average value was selected (as shown in Formula (1)).

(1) $$TBARS\left(mg\frac{MDA}{kg}\right)=\frac{c\times V\times 1000}{m\times 1000}$$

where: *c* represents the concentration of malondialdehyde in the sample solution obtained from the standard series curves/(μg/mL); *V* stands for constant volume of sample solution/mL; *m* represents the sample mass (g).

#### 2.3.4. Determination of Total Volatile Basic Nitrogen (TVB-N)

The determination of TVB-N referred to the method by Yi et al. [16]. TVB-N content was measured by distillation after addition of MgO (0.2 g) to the samples (2.0 g) in a Kjeldahl distilling flask (Kjeltec 8400, FOSS, Hiller, Denmark, FOSSScino (Suzhou) Co., Ltd., Suzhou, China). TVB-N content was expressed as mg of nitrogen per 100 g of sample.

#### 2.3.5. Determination of pH

A calibrated portable pH meter (Testo735-2, Testo AG, Lenzkirch, Germany) was used, and the pH probe was inserted into the fish after the temperature had been adjusted to the actual temperature of the samples. Each sample was measured at three different locations, and the average value was used as the final result [15].

#### 2.3.6. Determination of Texture

The textukral characteristics of the samples were determined by a textural instrument (CT3-10K, Brookfield Corporation, Middleboro, MA, USA). The test type was TPA texture analysis, the test target type was distance, the target value was 2.0 mm, the waiting time was 0 s, the trigger point load was 1.00 N, and the test speed was 1.00 mm/s. The probe model was TA18, cycle number was 2. The hardness, cohesion, elasticity and chewability of the sample were all measured.

#### 2.3.7. Sensory Evaluation

The sensory evaluation of blue round scad processed with red vinasse was carried out according to the scoring criteria in Table 1. The different products were numbered randomly and distributed to a panel of 10 sensory evaluators trained and experienced in sensory evaluations, with a 1:1 ratio of males to females [17]. The indexes of color, fragrance and tissue of the samples were evaluated.

### 2.4. Construction of Storage Quality Dynamic Model for Blue Round Scad Processed with Red Vinasse

First-order kinetic equation: Most changes in food processing follow the zero-order or first-order reaction model, and the first-order reaction kinetic model is widely used [18]. The first-order dynamic equation can reflect the relationship between the change in the storage quality index and time t, and can also predict the shelf life of products. The first-order reaction kinetics Equation (2) is as follows:

A = A_0_ekt (2)

where A is the observed quality index value until day t of storage; A_0_ is the observed quality index value at d 0 storage; k is the change rate constant of the storage quality index; t is the storage time (d) of the sample.

Arrhenius equation: The Arrhenius equation reflects the relationship between the rate constant k and the thermodynamic temperature T. After calculating the rate constant under different temperature conditions, a linear equation with slope-*Ea*/R and y-intercept lnk_0_ can be fitted by plotting 1/T with lnk, and the activation energy *Ea* and pre-factor k_0_ can be calculated. The Arrhenius Equation (3) is as follows:

(3) $$k=k_{0}\times exp(-\frac{Ea}{RT})$$

After taking the logarithm of the above formula:

(4) $$lnk=lnk_{0}-\frac{Ea}{RT}$$

where k_0_ is the pre-factor (frequency factor) of the equation; *Ea* is the activation energy/(J/moL) of the reaction to the change in storage quality index; T is the absolute temperature (K); R is the gas constant (8.314 4 J/(mol·K)). Both k_0_ and *Ea* were empirical constants related to the material nature of the reaction system.

When combining the first-order kinetic equation with the Arrhenius equation, the shelf life of the product can be predicted as long as the storage quality index value corresponding to the starting point and the end point of the sensory evaluation and a certain storage temperature is determined.

### 2.5. Statistical Analysis

The experiment was repeated three times, and the results were expressed as mean ± standard variance. ORIGIN 8.5 (OriginLab Corp., Northampton, MA, USA) was used for mapping, and SPSS Statistics 24.0 (IBM^®^ SPSS Statistics, Armonk, NY, USA) was used to test the significant differences between the results (*p* < 0.05) and multiple comparisons. The PLSR was analyzed using Unscrambler 9.7 software (CAMO ASA, Trondheim, Norway), and all data were standardized using SPSS 24.0 software prior to analysis.

## 3. Results and Discussion

### 3.1. Analysis of Physical and Chemical Indexes of Blue Round Scad Processed with Red Vinasse During Storage

#### 3.1.1. Changes in Fat Content of Blue Round Scad Processed with Red Vinasse During Storage

As can be seen from Table 2, the fat content of blue round scad samples showed a decreasing trend during the whole storage process (*p* < 0.05), and the fat content in samples from the experimental group (EG) of blue round scad processed with red vinasse was higher than that in samples from the control group (CG) of blue round scad not processed with red vinasse. The fat content in EG samples decreased faster in the early stage of storage than in the later stage. This may be due to the fact that some changes had taken place between the complex components in the red vinasse and the chemical components of the fish flesh during the red vinasse processing, and the existence of some antioxidant components in the red vinasse played a certain role in inhibiting lipid oxidation [3,19]. In addition, the decrease in water content in the fish samples at the later stage of storage also could have slowed the rate of fat decomposition [5,6].

#### 3.1.2. Change in TBARS

**Figure 1:**
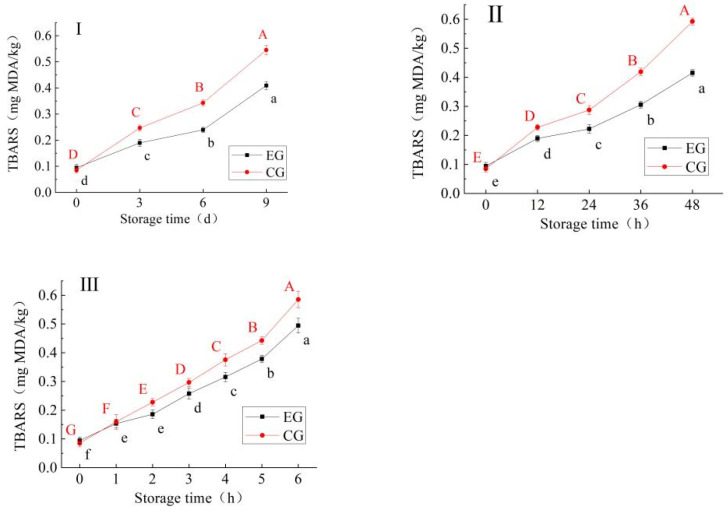
Change in TBARS in blue round scad samples during storage at 4 °C (**I**), 25 °C (**II**), 37 °C (**III**) (EG: the experimental group of blue round scad samples processed with red vinasse; CG: the control group of blue round scad samples not processed with red vinasse; a–f: different lowercase letters represented significant differences between EG data (*p* < 0.05); A–G: different capital lettersdata represented significant differences between CG data (*p* < 0.05)).

The TBARS value is positively correlated with the degree of lipid oxidation and is the main index to directly reflect the final product of lipid oxidation. Studies have shown that the higher the TBARS value, the higher the degree of fat oxidation and the worse the product quality [20]. The recommended limit standard of the TBARS value is 0.5 mg MDA/kg in the literature [21,22]. As can be seen from Figure 1, with the extension of storage time, the TBARS values of the samples in all groups were significantly increased (*p* < 0.05). On the whole, the TBARS values for EG samples were lower than those for the CG samples during storage, and increased more significantly in CG samples at the later stage of storage (*p* < 0.05). The samples of CG exceeded the critical value of TBARS before the samples of EG. The results showed that processing with red vinasse could inhibit the oxidation of lipids in blue round scad. This may be due to the fact that some active ingredients (such as the polyphenols) in the red vinasse can inhibit the production of malondialdehyde during storage [23,24].

#### 3.1.3. Changes in PUFA Composition

Blue round scad is delicious and nutritious, and the fat is rich in PUFAs, especially n-3 PUFAs such as eicosapentaenoic acid (EPA) and docosahexaenoic acid (DHA) [25,26]. PUFAs have important physiological functions for the human body. They can treat and prevent cardiovascular and cerebrovascular diseases, regulate lipid metabolism, and can be used for anti-cancer, anti-aging, beauty and other applications [27]. However, it has also been proved that the highly unsaturated structure of PUFAs is easily affected by light, oxygen and heat, resulting in poor storage capacity and unstable functional characteristics of blue round scad [28].

By GC analysis, 22 types of fatty acids in blue round scad were identified, and PUFAs ere mainly composed of eicosapentaenoic acid (C20:4n-6), EPA, docosapentaenoic acid (DPA), DHA, etc. The changes in PUFA composition at 4 °C, 25 °C and 37 °C are shown in Table 3, Table 4 and Table 5, respectively. Under different storage temperatures, PUFAs in EG and CG showed an overall downward trend, and the decline in the CG samples was more significant (*p* < 0.05). During storage at 4 °C, the EG samples showed the largest decrease in 0–3 d, especially the C20:4n-6 and DHA, which are important for the nutritional quality of seafood and have many health benefits (Table 3). During storage at 25 °C, PUFAs in EG and CG decreased the most within 36–48 h, during the later stage of storage (Table 4). During storage at 37 °C, PUFAs decreased more evenly between EG and CG (Table 5). On the whole, the range of variation in PUFAs in CG samples was significantly greater than that in the EG samples, indicating that the processing with red vinasse could slow down the oxidation rate of PUFA components, which was consistent with the results in Section 3.1.1 and Section 3.1.2.

**Figure 2:**
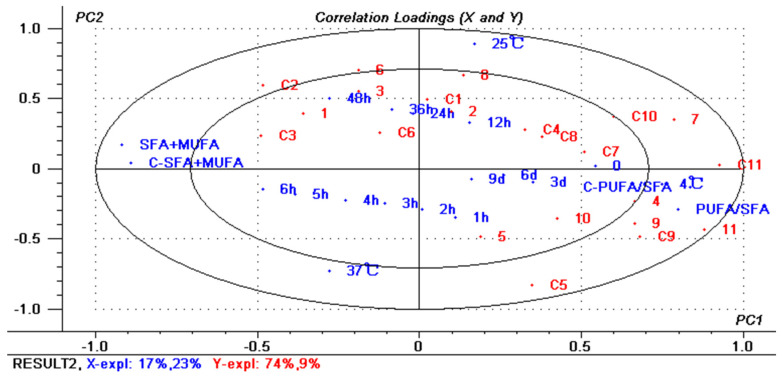
Loading plots of correlation of each index. The *X*-axis represents the main design variables: 17 of 0/1 sample variables (3 storage temperatures, 14 storage times), PUFA/SFA values, and SFA + MUFA values. The *Y*-axis represents PUFA composition. Numbers 1 to 22 represent C18:2n-6 to C22:6n-3 of EG, respectively. Numbers C1~C11 represent C18:2n-6c~C22:6n-3 of CG, respectively. The inner and outer circles represent correlation coefficients r^2^ = 0.5 (50%) and r^2^ = 1.0 (100%), respectively.

In order to more clearly describe the effects of different storage temperatures and durations on the changes in PUFA composition in blue round scad processed with red vinasse, partial least squares regression (PLSR) was used to establish a mathematical model, and the load diagram is shown in Figure 2. The horizontal axis represents the score of the first principal component of the sample, and the vertical axis represents the score of the second principal component of the sample. The main factor 1 explained 17% of the total variation, and the main factor 2 explained 23% of the total variation. Principal components 1 and 2 explained 74% and 9% of the Y variable, respectively. It can be seen that the PUFA composition in blue round scad processed with red vinasse changed mainly in the first principal component during storage.

It was reported that the PUFA/SFA value is generally used to evaluate the nutritional value of meat, and the higher the value, the better the nutritional value. At the same time, the higher the SFA + MUFA value (1-PUFA%), the better the tenderness, juiciness and flavor of meat products, so the SFA + MUFA value (1-PUFA%) can be used to measure the flavor and other related quality indicators after food processing [14,29]. Therefore, according to the changes in the composition of PUFAs and (1-PUFA%) at different storage temperatures, we can conclude that the sample closer to the right side of the loading diagram had better nutritional value in terms of fatty acids, while the sample closer to the left side of the loading diagram had better tenderness and juiciness. Under the storage conditions of 4 °C and 25 °C, the variations in PUFA composition in blue round scad samples were more similar, and the lower the temperature, the better the nutritional composition of fatty acids. With the extension of storage time, the sum of SFA and MUFA in the samples increased (1-PUFA%), that is, the PUFA content gradually decreased.

In terms of the changes in PUFAs, 11 (C22:6n-3) and 9 (C20:5n-3) in EG and CG were very close to the first principal component of PUFA/SFA, indicating that they both had high relative content in the early storage stage, which is an important component affecting the nutritional composition of fatty acids in the EG samples. Similarly, 4 (20:2n-6) and 7 (C20:4n-6) in EG also had important effects on the changes in fatty acid nutrition and PUFA composition during storage and had higher relative contents than those in CG. In EG, 1 (C18:2n-6), 3 (C18:3n-3) and 6 (C20:3n-3) were closer to SFA + MUFA and had less relative content than other PUFAs, which had little effect on the nutritional composition of fatty acids during storage, and the content showed a significant trend of increase. In CG, 2 (C18:3n-6) and 3 (C18:3n-3) also showed an overall increasing trend, which was significantly different from the changes in other fatty acids.

#### 3.1.4. Changes in TVB-N

**Figure 3:**
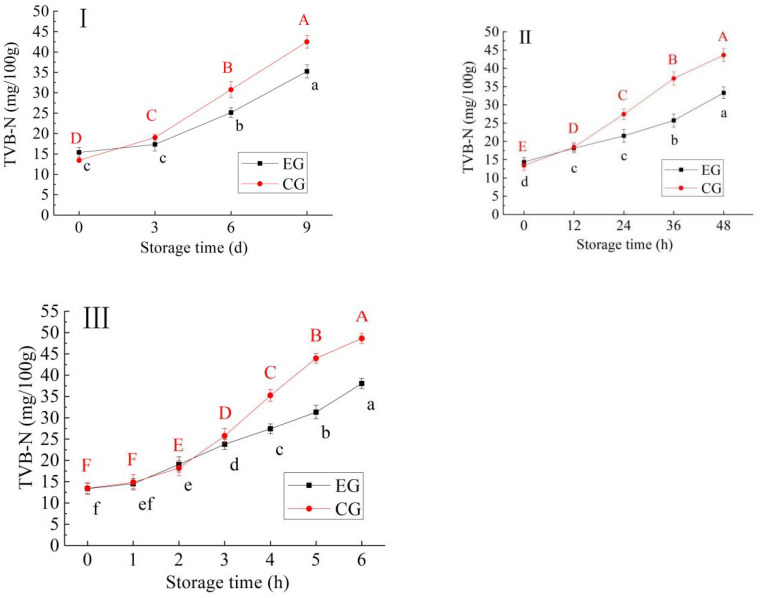
Change in TVB-N in blue round scad samples during storage at 4 °C (**I**), 25 °C (**II**), 37 °C (**III**) (EG: the experimental group of blue round scad samples processed with red vinasse; CG: the control group samples of blue round scad not processed with red vinasse; a–f: different lowercase letters represented significant differences between EG data (*p* < 0.05); A–F: different capital lettersdata represented significant differences between CG data (*p* < 0.05)).

As can be seen from Figure 3, the TVB-N value in EG and CG samples showed a significant increasing trend (*p* < 0.05). This may be because with the extension of storage time, the microorganisms in fish will accelerate the decomposition of fish proteins and produce alkaline substances such as ammonia and amines, so the TVB-N value of fish meat will increase during storage [15]. At the same time, the TVB-N value of the EG samples increased more slowly than that of the CG samples, which may be because the *monascus* and its metabolites inhibited the growth of spoilage bacteria in the fish and thus reduced the production rate of TVB-N [23]. Different foods have different thresholds of TVB-N. Generally speaking, a TVB-N value below 15 mg/100 g indicates freshness, 15–30 mg/100 g indicates the beginning of spoilage, and more than 30 mg/100 g indicates a spoiled condition [30]. Therefore, at 4, 25 and 37 °C, the samples in EG were storable roughly for 8 d, 48 h, and 5 h, while the samples in CG were storable for 6 d, 30 h, and 3.5 h, respectively.

#### 3.1.5. Changes in pH Value

**Figure 4:**
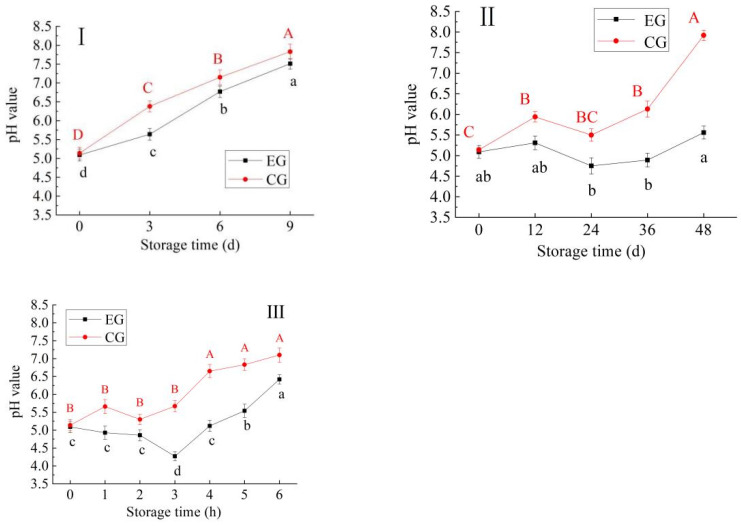
Change in pH of blue round scad samples during storage at 4 °C (**I**), 25 °C (**II**), 37 °C (**III**) (EG: the experimental group of blue round scad samples processed with red vinasse; CG: the control group of blue round scad samples not processed with red vinasse; a–d: different lowercase letters represented significant differences between EG data (*p* < 0.05); A–D: different capital lettersdata represented significant differences between CG data (*p* < 0.05)).

Changes in the pH of blue round scad samples during storage at 4 °C, 25 °C and 37 °C are shown in Figure 4. At 4 °C, the pH value of blue round scad samples showed an overall rising trend, while at 25 °C and 37 °C, the pH value showed a trend of first decreasing and then increasing, and the pH of CG samples was significantly higher than that of EG samples during the whole storage period (*p* < 0.05). The reason for this phenomenon may be that the red vinasse itself was weakly acidic, and there will be a small amount of fermentation, producing acid at 25 °C and 37 °C. However, with the extension of storage time, proteins and other nitrogen-containing substances in the fish will be decomposed by microorganisms into alkaline substances such as ammonia, trimethyl amine and indole, and the red vinasse may increase free amino acid content and slow down the rancidity of fish lipids as well, resulting in a gradual increase in the pH value of the fish [31,32]. The results were consistent with those reported by Zeng et al. [33]. In the early storage period, the pH of fish meat may be acidic due to the anaerobic glycolysis of fish meat and the degradation of ATP, producing inorganic phosphoric acid; in the later storage period, due to the microbial degradation of proteins in fish meat and the continuous accumulation of alkaline substances such as trimethylamine and ammonia, the pH gradually increases [34].

#### 3.1.6. Changes in Texture Properties

The values of various structural properties determined during different storage periods of samples of EG and CG are shown in Table 6. During storage, the hardness value, cohesion and chewiness of samples in each group showed a decreasing trend (*p* < 0.05), which may be due to the decomposition and destruction of muscle fiber tissue and protein structure under the action of microorganisms and enzymes in fish. In addition, it can be seen that the hardness, cohesion and chewiness values of the EG samples were larger than those of the CG samples, while the elasticity value was smaller than that of the CG samples (*p* < 0.05). As reported in the literature, the texture properties of fish are determined by several internal factors, such as the density of muscle fibers and the content of collagen [35], and the change in texture characteristics during cold storage is mainly related to water content and water distribution [36]. Therefore, it may be that the red vinasse processing can slow down the destruction of fish protein and muscle fiber tissue caused by microorganisms and enzymes. The study of Benito et al. showed that the denaturation of protein, acid gel and dehydration of meat products during fermentation can enhance the texture of meat products, which was basically consistent with the experimental results [37].

### 3.2. Changes in Sensory Score

**Figure 5:**
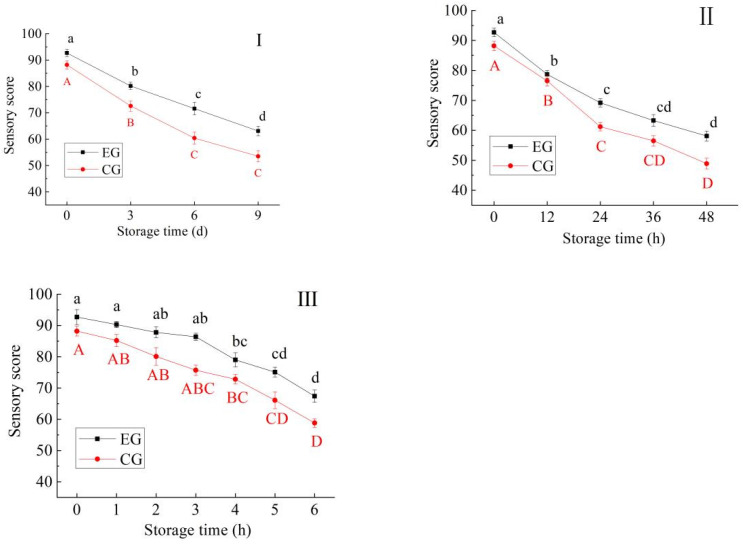
Change in sensory score for blue round scad samples during storage at 4 °C (**I**), 25 °C (**II**), and 37 °C (**III**) (EG: the experimental group of blue round scad samples processed with red vinasse; CG: the control group of blue round scad samples not processed with red vinasse; a–d: different lowercase letters represented significant differences between EG data (*p* < 0.05); A–D: different capital lettersdata represented significant differences between CG data (*p* < 0.05)).

As can be seen from Figure 5, the sensory scores for blue round scad samples in all groups showed a downward trend with the extension of storage time at 4 °C (I), 25 °C (II), and 37 °C (III), and the decline was more obvious in CG (*p* < 0.05). This may be due to the continuous oxidation and degradation of proteins in fish, resulting in the destruction of muscle structure, while generating trimethylamine, humutine and other odorous substances [33,34]. However, the processing with red vinasse can slow down the above quality deterioration, as verified by the results in Section 3.1 and Section 3.2. Therefore, processing with red vinasse can improve the color, flavor and fish structure of blue round scad to a certain extent, and also can slow down the decline in sensory quality caused by spoilage.

### 3.3. Establishment and Verification of Dynamic Model of Quality Changes in Blue Round Scad Processed with Red Vinasse During Storage

#### 3.3.1. Correlation Between Sensory Evaluation and Physicochemical Indexes of Blue Round Scad Processed with Red Vinasse at Different Storage Temperatures

As can be seen from Table 7, the sensory evaluation scores for blue round scad samples in the two groups (EG and CG) showed a good correlation with the changes in various physical and chemical indexes (*p* < 0.05), and most of the Pearson correlation coefficients were greater than 0.9. Among them, the correlation coefficient for the TBARS value was high and very significant. This may be because the TBARS value is closely related to lipid oxidation in fish; especially in the presence of water, fish with higher PUFA content is prone to spoilage, which is manifested by significant changes in texture, color, and related physicochemical indexes [20,21,22]. In view of this, the value of TBARS was selected to construct a shelf life prediction model during storage of blue round scad processed or not processed with red vinasse.

#### 3.3.2. Regression Equation of Change in TBARS Value for EG Samples Under Different Storage Temperatures

By using Origin 8.5 software, the variation curves for TBARS values under different storage temperatures were linearly fitted, and the fitting linear regression equations, regression coefficients R^2^ and rate constants k were obtained. As can be seen from Table 8, the multiple correlation coefficient R^2^ values for the regression equations established at storage temperatures of 4 °C (277.15 K), 25 °C (298.15 K) and 37 °C (310.15 K) were approximately or greater than 0.95, indicating that the regression equations had a high degree of fit. At the same time, the rate constants k of TBARS were 0.00138, 0.00632 and 0.06356, respectively.

The linear equation y = −9.4251X + 27.20091 was obtained by plotting lnk to the inverse of the storage temperature (1/T). The activation energy *Ea* with TBARS value was 7.8364 × 10^4^ J/mol, and the pre-factor k_0_ was 6.5044 × 10^11^. On this basis, the Arrhenius equation, kinetic equation and shelf life prediction equation between the change rate constant k of the TBARS value and storage temperature T in EG were established as follows: Arrhenius Equation (5):

(5) $$A=A_{0}\times e^{(\frac{-7.8364\times 10^{4}}{8.3144T})t}$$

First-order kinetic equation:

(6) $$k=650436816816.915\times e^{\frac{-7.8364\times {\;10}^{4}}{8.3144T}}$$

Formula of shelf life prediction:

(7) $$SL=\frac{\ln\left(TBARS\right)-ln({TBARS}_{0})}{650436816816.915\times exp(\frac{-7.8364\times 10^{4}}{8.3144T})}$$

#### 3.3.3. Regression Equation of Change in TBARS Value for CG Samples Under Different Storage Temperatures

The linear regression equation, regression coefficient R^2^ and the change rate constant k were obtained by using Origin to fit the change curve for the TBARS value under different storage temperatures. As can be seen from Table 9, the multiple correlation coefficients R^2^ of the regression equations established at different storage temperatures of 4 °C (277.15 K), 25 °C (298.15 K) and 37 °C (310.15 K) were all greater than 0.95, indicating that the regression equations had a high degree of fit. At the same time, the rate constants k of TBARS were 0.00205, 0.01005 and 0.07905, respectively.

The linear equation y = −9.0633x + 26.33156 was obtained by plotting the inverse of lnk to storage temperature 1/T. The activation energy *Ea* with peroxide value was 7.5356 × 10^4^ J/mol, and the pre-factor k_0_ was 2.7268 × 10^11^. On this basis, the Arrhenius equation, kinetic equation and shelf life prediction equation between the change rate constant k of TBARS value and storage temperature T in CG were established as follows:

Arrhenius equation:

(8) $$A=A_{0}\times e^{(\frac{-7.5356\times 10^{4}}{8.3144T})t}$$

First-order kinetic equation:

(9) $$k=272678695654.429\times e^{\frac{-7.5356\times {\;10}^{4}}{8.3144T}}$$

Formula of shelf life prediction:

(10) $$SL=\frac{\ln\left(TBARS\right)-ln({TBARS}_{0})}{272678695654.429\times exp(\frac{-7.5356\times {\;10}^{4}}{8.3144T})}$$

According to the equation for shelf life prediction with the TBARS value obtained above, when the storage temperature, initial TBARS value and end TBARS value are determined, the storage time for blue round scad can be calculated under a given temperature condition, such that the shelf life of blue round scad processed or not processed with red vinasse can be predicted. In addition, to monitor the change in product quality according to the constructed shelf life prediction model, the storage temperature, initial TBARS value and storage time can be used to calculate the TBARS value after a certain storage temperature condition [15,38].

#### 3.3.4. Dynamic Model for Prediction of Quality Changes in Fish at Different Storage Temperatures

According to the recommended limit standard TBARS value of 0.5 mg MDA/kg in the literature [21,22], the predicted shelf life values for EG samples were 55 d, 2.7 d and 28 h at the storage temperatures of 4 °C, 25 °C and 37 °C, respectively. The predicted shelf-life values for CG samples were 38 d, 1.5 d and 20 h, respectively, and the prediction errors were all less than 10%. The experimental results showed that the actual shelf life of EG samples was 50 d, 2.5 d and 26 h at 4 °C, 25 °C and 37 °C, respectively, which was significantly longer than that of the CG samples (36 d, 1.4 d and 19 h) (Table 10), indicating that the predicted shelf life of the EG samples was longer than that of the CG samples, that is, the processing with red vinasse could prolong the shelf life of blue round scad. It was speculated that this may be related to *monascus* and its metabolites in red vinasse, such as polyphenols, *monascus* pigment and lovastatin, which have antibacterial, anticorrosive and antioxidant properties [3,39].

## 4. Conclusions

The red vinasse processing had a significant effect on the quality of blue round scad samples during storage.

During different storage periods, the red vinasse processing can significantly inhibit fat oxidation, reduce the production of TBARS in fish, maintain a high relative content of PUFAs, reduce the production of TVB-N, slow down the rate of sensory quality decline, and maintain good texture qualities of fish. Correlation analysis between sensory scores and changes in physical and chemical indexes at different storage temperatures found that the correlation coefficient between sensory scores and TBARS value was as high as 0.996. A regression equation based on changes in TBARS value was established through a first-order kinetic model combined with the Arrhenius equation, and the shelf life of the blue round scad samples in EG was significantly longer than that of the CG samples (38 d, 1.5 d and 20 h). In summary, the red vinasse processing can improve the physical and chemical quality of blue round scad during storage and prolong the shelf life of the product. The results of this study will provide a certain theoretical reference for the development and innovation of Fujian traditional red vinasse foods, as well as the safe storage of blue round scad.

## Figures and Tables

**Table 1:** Scoring criteria for the samples.

Index	Scoring Standard	Score
Color (30)	The color is bright and red.	26~30
	The color is bright red and the brightness is general.	21~25
	The color is relatively dark red.	16~20
	The color is dark red.	10~15
Fragrance (40)	Weak fishy taste, no odor, experimental group fish mellow.	36~40
	The fishy taste was more obvious, no odor, and the fish of the experimental group was mellow.	31~35
	The smell of fish is obvious, there is a weak smell of corruption, the experimental group fish is light mellow.	26~30
	The fishy taste was obviously accompanied by serious spoilage odor, and the fish in the experimental group had no aroma.	20~25
Tissue (30)	The meat is firm and elastic with no mucous surface.	26~30
	The flesh is firm and still elastic, with no mucous surface.	21~25
	The meat is loose, basically inelastic, with a small amount of mucus on the surface.	16~20
	The flesh is loose, inelastic, and the surface slime is obvious.	10~15

**Table 2:** Changes in fat content of blue round scad processed with red vinasse during storage (%).

Storage Temperature (°C)	Storage Time	Fat Content (%)	
		EG	CG
4	0 d	4.88 ± 2.19 ^a^	3.48 ± 0.86 ^a^
	3 d	3.82 ± 0.98 ^b^	2.19 ± 0.48 ^b^
	6 d	2.43 ± 2.77 ^c^	1.67 ± 1.24 ^c^
	9 d	2.36 ± 0.91 ^c^	1.38 ± 0.41 ^d^
25	0 h	4.88 ± 2.19 ^a^	3.48 ± 0.86 ^a^
	12 h	3.78 ± 0.72 ^b^	3.16 ± 0.20 ^b^
	24 h	2.54 ± 0.18 ^c^	2.74 ± 0.59 ^c^
	36 h	2.82 ± 1.50 ^c^	1.97 ± 0.25 ^d^
	48 h	2.02 ± 1.19 ^d^	1.77 ± 0.35 ^e^
37	0 h	4.88 ± 2.19 ^a^	3.48 ± 0.86 ^a^
	1 h	4.94 ± 0.47 ^a^	3.35 ± 0.88 ^a^
	2 h	4.01 ± 0.91 ^b^	2.69 ± 1.17 ^b^
	3 h	3.29 ± 1.87 ^c^	2.49 ± 0.52 ^c^
	4 h	3.37 ± 0.81 ^c^	2.18 ± 0.07 ^d^
	5 h	2.73 ± 0.31 ^d^	1.49 ± 1.16 ^f^
	6 h	2.12 ± 1.05 ^e^	1.80 ± 0.81 ^e^

Note: Different letters in the same column at each storage temperature represent significant differences between the results (*p* < 0.05). EG: the experimental group of blue round scad samples processed with red vinasse; CG: the control group of blue round scad samples not processed with red vinasse.

**Table 3:** Change in PUFA composition in blue round scad during storage at 4 °C (%).

No.	Composition of PUFA	Name	EG				CG			
			0 d	3 d	6 d	9 d	0 d	3 d	6 d	9 d
1	C18:2n-6c	LA	1.20 ± 0.02 ^c^	0.80 ± 0.00 ^d^	1.69 ± 0.02 ^a^	1.40 ± 0.00 ^b^	0.83 ± 0.03 ^b^	0.76 ± 0.00 ^c^	0.87 ± 0.01 ^b^	1.04 ± 0.01 ^a^
2	C18:3n-6	γ-LA	0.57 ± 0.01 ^b^	0.64 ± 0.00 ^a^	0.56 ± 0.01 ^b^	0.53 ± 0.00 ^d^	0.37 ± 0.01 ^b^	0.41 ± 0.00 ^a,b^	0.37 ± 0.00 ^b^	0.42 ± 0.03 ^a^
3	C18:3n-3	ALA	1.09 ± 0.05 ^b^	1.29 ± 0.00 ^a^	1.07 ± 0.01 ^b^	0.96 ± 0.00 ^d^	0.95 ± 0.02 ^a^	0.87 ± 0.00 ^b^	0.92 ± 0.01 ^a^	0.92 ± 0.02 ^a^
4	C20:2n-6	EDA	0.56 ± 0.02 ^a^	0.42 ± 0.02 ^b^	0.26 ± 0.00 ^d^	0.25 ± 0.00 ^d^	0.47 ± 0.03 ^a^	0.24 ± 0.00 ^d^	0.29 ± 0.01 ^c^	0.40 ± 0.01 ^b^
5	C20:3n-6	DGLA	3.53 ± 0.02 ^a^	3.49 ± 0.01 ^a^	2.93 ± 0.04 ^d^	3.15 ± 0.01 ^c^	3.09 ± 0.00 ^b^	3.46 ± 0.00 ^a^	2.91 ± 0.03 ^c^	3.43 ± 0.01 ^a^
6	C20:3n-3	ETA	0.24 ± 0.04 ^c^	0.27 ± 0.00 ^c^	0.51 ± 0.01 ^a^	0.44 ± 0.01 ^b^	0.30 ± 0.01 ^c^	0.39 ± 0.04 ^b^	0.42 ± 0.00 ^a,b^	0.45 ± 0.00 ^a^
7	C20:4n-6	AA	9.24 ± 0.17 ^a^	7.80 ± 0.00 ^b^	7.47 ± 0.17 ^b^	6.53 ± 0.09 ^d^	8.89 ± 0.23 ^a^	6.73 ± 0.04 ^c^	7.82 ± 0.01 ^b^	5.74 ± 0.01 ^d^
8	C22:2n-6	DDAn-6	0.43 ± 0.04 ^c^	0.51 ± 0.00 ^b^	0.47 ± 0.01 ^b,c^	0.78 ± 0.00 ^a^	0.34 ± 0.01 ^b^	0.71 ± 0.00 ^a^	0.61 ± 0.21 ^a,b^	0.55 ± 0.01 ^a,b^
9	C20:5n-3	EPA	2.36 ± 0.14 ^a,b^	2.32 ± 0.00 ^a,b^	2.22 ± 0.04 ^b^	2.49 ± 0.02 ^a^	2.17 ± 0.04 ^c^	2.72 ± 0.01 ^a^	2.35 ± 0.09 ^b^	2.12 ± 0.01 ^c^
10	C22:5n-3	DPA	2.56 ± 0.01 ^a^	1.87 ± 0.01 ^b^	2.58 ± 0.04 ^a^	2.57 ± 0.01 ^a^	2.65 ± 0.10 ^a,b^	2.72 ± 0.01 ^a^	2.69 ± 0.02 ^a^	2.53 ± 0.01 ^b^
11	C22:6n-3	DHA	33.26 ± 0.04 ^a^	33.04 ± 0.01 ^a^	31.82 ± 0.17 ^b^	31.69 ± 0.05 ^b^	34.22 ± 0.07 ^a^	32.01 ± 0.03 ^b^	30.62 ± 0.14 ^c^	29.12 ± 0.10 ^d^
	PUFA		55.03 ± 0.07 ^a^	52.44 ± 0.03 ^b^	51.59 ± 0.14 ^c^	50.79 ± 0.03 ^d^	54.28 ± 0.11 ^a^	51.01 ± 0.04 ^b^	49.86 ± 0.06 ^c^	46.73 ± 0.09 ^d^
	SFA		31.57 ± 0.01 ^d^	35.88 ± 0.04 ^c^	37.80 ± 0.18 ^b^	39.20 ± 0.05 ^a^	31.99 ± 0.14 ^d^	37.29 ± 0.02 ^c^	40.09 ± 0.15 ^b^	44.04 ± 0.07 ^a^
	MUFA		13.40 ± 0.06 ^a^	11.68 ± 0.07 ^b^	10.62 ± 0.32 ^c^	10.01 ± 0.08 ^d^	13.73 ± 0.25 ^a^	11.70 ± 0.06 ^b^	10.05 ± 0.08 ^c^	9.23 ± 0.01 ^d^

Note: Different letters in the same row represent significant differences between the results (*p* < 0.05), the same below. LA: Linoleic acid; γ-LA: γ-Linolenic acid; ALA: α-Linolenic acid; EDA: Eicosadienoic acid; DGLA: 8,11,14-Eicosatrienoic acid; ETA: Eicosatrienoic acid; AA: Arachidonic acid; DDAn-6: Docosahrodienoic acid; EPA: Eicosapentaenoic acid; DPA: Docosapentaenoic acid; DHA: Docosahexaenoic acid; PUFA: Polyunsaturated fatty acid; SFA: Saturated fatty acid; MUFA: Monounsaturated fatty acid.

**Table 4:** Change in PUFA composition in blue round scad during storage at 25 °C (%).

No.	Composition of PUFA	Name	EG					CG				
			0 h	12 h	24 h	36 h	48 h	0 h	12 h	24 h	36 h	48 h
1	C18:2n-6c	LA	1.20 ± 0.02 ^d^	1.20 ± 0.02 ^d^	1.46 ± 0.01 ^b^	1.41 ± 0.00 ^c^	1.64 ± 0.01 ^a^	0.83 ± 0.03 ^c^	1.05 ± 0.01 ^b^	1.22 ± 0.01 ^a^	1.05 ± 0.01 ^b^	1.05 ± 0.00 ^b^
2	C18:3n-6	γ-LA	0.57 ± 0.01 ^b^	0.61 ± 0.04 ^b^	0.59 ± 0.01 ^b^	0.56 ± 0.02 ^b^	0.76 ± 0.01 ^a^	0.37 ± 0.01 ^c^	0.56 ± 0.00 ^b^	0.56 ± 0.01 ^b^	0.58 ± 0.00 ^b^	0.69 ± 0.06 ^a^
3	C18:3n-3	ALA	1.09 ± 0.05 ^b^	1.74 ± 0.01 ^a^	1.82 ± 0.11 ^a^	1.79 ± 0.09 ^a^	1.80 ± 0.01 ^a^	0.95 ± 0.02 ^e^	1.25 ± 0.01 ^d^	1.43 ± 0.01 ^a^	1.39 ± 0.00 ^b^	1.33 ± 0.02 ^c^
4	C20:2n-6	EDA	0.56 ± 0.02 ^a^	0.23 ± 0.06 ^b^	0.21 ± 0.03 ^b^	0.24 ± 0.08 ^b^	0.26 ± 0.01 ^b^	0.47 ± 0.03 ^a^	0.24 ± 0.02 ^c^	0.24 ± 0.00 ^c^	0.40 ± 0.00 ^b^	0.42 ± 0.05 ^a,b^
5	C20:3n-6	DGLA	3.53 ± 0.02 ^a^	3.42 ± 0.04 ^b^	3.21 ± 0.01 ^c^	3.13 ± 0.01 ^d^	3.05 ± 0.02 ^e^	3.09 ± 0.00 ^a^	2.59 ± 0.01 ^b^	2.15 ± 0.01 ^c^	1.95 ± 0.00 ^d^	1.91 ± 0.01 ^e^
6	C20:3n-3	ETA	0.24 ± 0.04 ^c^	0.58 ± 0.02 ^b^	0.64 ± 0.00 ^a^	0.57 ± 0.00 ^b^	0.60 ± 0.00 ^a,b^	0.30 ± 0.01 ^b^	0.43 ± 0.03 ^a^	0.44 ± 0.10 ^a^	0.40 ± 0.00 ^a,b^	0.49 ± 0.00 ^a^
7	C20:4n-6	AA	9.24 ± 0.17 ^a^	8.50 ± 0.02 ^b^	8.03 ± 0.00 ^c^	7.56 ± 0.01 ^d^	6.93 ± 0.04 ^e^	8.89 ± 0.23 ^a^	8.65 ± 0.01 ^a^	7.58 ± 0.08 ^b^	7.56 ± 0.00 ^b^	6.22 ± 0.07 ^c^
8	C22:2n-6	DDAn-6	0.43 ± 0.04 ^c^	0.93 ± 0.00 ^a^	0.78 ± 0.01 ^a,b^	0.90 ± 0.01 ^a^	0.72 ± 0.14 ^b^	0.34 ± 0.01 ^d^	0.80 ± 0.00 ^a^	0.45 ± 0.00 ^c^	0.60 ± 0.00 ^b^	0.46 ± 0.01 ^c^
9	C20:5n-3	EPA	2.36 ± 0.14 ^a^	1.93 ± 0.03 ^b^	2.01 ± 0.00 ^b^	1.82 ± 0.01 ^a,b^	1.65 ± 0.15 ^c^	2.17 ± 0.04 ^b^	2.25 ± 0.03 ^a^	1.98 ± 0.00 ^c^	1.89 ± 0.01 ^d^	1.42 ± 0.03 ^e^
10	C22:5n-3	DPA	2.56 ± 0.01 ^a^	2.56 ± 0.02 ^a^	2.20 ± 0.02 ^c^	2.29 ± 0.01 ^b^	1.84 ± 0.05 ^d^	2.65 ± 0.10 ^a^	2.61 ± 0.01 ^a^	2.65 ± 0.00 ^a^	2.57 ± 0.02 ^a^	2.30 ± 0.04 ^b^
11	C22:6n-3	DHA	33.26 ± 0.04 ^a^	31.07 ± 0.11 ^b^	28.99 ± 0.11 ^c^	28.11 ± 0.04 ^d^	25.30 ± 0.00 ^e^	34.22 ± 0.07 ^a^	31.70 ± 0.37 ^b^	29.74 ± 0.13 ^c^	29.14 ± 0.01 ^d^	26.42 ± 0.28 ^e^
	PUFA		55.03 ± 0.07 ^a^	52.78 ± 0.03 ^b^	49.95 ± 0.06 ^c^	48.40 ± 0.09 ^d^	44.54 ± 0.16 ^e^	54.28 ± 0.11 ^a^	52.14 ± 0.39 ^b^	48.45 ± 0.34 ^c^	47.54 ± 0.06 ^d^	42.73 ± 0.47 ^e^
	SFA		31.57 ± 0.01 ^e^	34.08 ± 0.02 ^d^	37.37 ± 0.01 ^c^	39.54 ± 0.05 ^b^	43.24 ± 0.16 ^a^	31.99 ± 0.14 ^e^	35.99 ± 0.27 ^d^	39.84 ± 0.40 ^c^	41.28 ± 0.12 ^b^	46.36 ± 0.10 ^a^
	MUFA		13.40 ± 0.06 ^a^	13.13 ± 0.02 ^b^	12.68 ± 0.05 ^c^	12.06 ± 0.05 ^d^	12.22 ± 0.00 ^e^	13.73 ± 0.25 ^a^	11.86 ± 0.11 ^b^	11.70 ± 0.07 ^b,c^	11.19 ± 0.06 ^c,d^	10.91 ± 0.37 ^d^

Note: Different letters in the same row represent significant differences between the results (*p* < 0.05), the same below. LA: Linoleic acid; γ-LA: γ-Linolenic acid; ALA: α-Linolenic acid; EDA: Eicosadienoic acid; DGLA: 8,11,14-Eicosatrienoic acid; ETA: Eicosatrienoic acid; AA: Arachidonic acid; DDAn-6: Docosahrodienoic acid; EPA: Eicosapentaenoic acid; DPA: Docosapentaenoic acid; DHA: Docosahexaenoic acid; PUFA: Polyunsaturated fatty acid; SFA: Saturated fatty acid; MUFA: Monounsaturated fatty acid.

**Table 5:** Change in PUFA composition in blue round scad during storage at 37 °C (%).

No.	Composition of PUFA	Name	EG							CG						
			0 h	1 h	2 h	3 h	4 h	5 h	6 h	0 h	1 h	2 h	3 h	4 h	5 h	6 h
1	C18:2n-6c	LA	1.20 ± 0.02 ^c^	1.21 ± 0.02 ^c^	0.93 ± 0.01 ^d^	1.37 ± 0.03 ^b^	1.36 ± 0.01 ^b^	1.21 ± 0.01 ^c^	1.50 ± 0.10 ^a^	0.83 ± 0.03 ^c^	1.19 ± 0.04 ^a^	0.81 ± 0.00 ^c^	0.90 ± 0.00 ^b^	0.84 ± 0.01 ^c^	0.89 ± 0.00 ^b^	0.74 ± 0.00 ^d^
2	C18:3n-6	γ-LA	0.57 ± 0.01 ^b,c^	0.42 ± 0.08 ^d^	0.58 ± 0.01 ^b,c^	0.71 ± 0.01 ^a^	0.62 ± 0.00 ^b^	0.35 ± 0.00 ^d^	0.51 ± 0.02 ^c^	0.37 ± 0.01 ^c^	0.41 ± 0.06 ^b,c^	0.53 ± 0.01 ^a^	0.57 ± 0.00 ^a^	0.52 ± 0.00 ^a^	0.45 ± 0.02 ^b^	0.44 ± 0.01 ^b^
3	C18:3n-3	ALA	1.09 ± 0.05 ^d^	0.53 ± 0.00 ^f^	1.63 ± 0.05 ^b^	1.76 ± 0.01 ^a^	1.56 ± 0.02 ^b,c^	1.46 ± 0.02 ^c^	0.67 ± 0.10 ^e^	0.95 ± 0.02 ^e^	0.59 ± 0.04 ^f^	1.53 ± 0.01 ^b^	1.74 ± 0.01 ^a^	1.30 ± 0.01 ^c^	1.27 ± 0.03 ^c^	1.14 ± 0.01 ^d^
4	C20:2n-6	EDA	0.56 ± 0.02 ^a^	0.35 ± 0.01 ^b^	0.24 ± 0.02 ^d^	0.33 ± 0.07 ^b,c^	0.27 ± 0.01 ^c,d^	0.25 ± 0.01 ^d^	0.22 ± 0.00 ^d^	0.47 ± 0.03 ^a^	0.36 ± 0.00 ^b^	0.26 ± 0.02 ^c,d^	0.27 ± 0.00 ^c^	0.26 ± 0.01 ^c,d^	0.27 ± 0.00 ^c^	0.24 ± 0.01 ^d^
5	C20:3n-6	DGLA	3.53 ± 0.02 ^c^	4.21 ± 0.13 ^a^	3.35 ± 0.03 ^d^	3.74 ± 0.03 ^b^	3.60 ± 0.06 ^b,c^	3.71 ± 0.05 ^b^	2.77 ± 0.04 ^e^	3.09 ± 0.00 ^d^	4.53 ± 0.04 ^a^	3.08 ± 0.00 ^d^	3.24 ± 0.01 ^b^	3.18 ± 0.03 ^c^	3.04 ± 0.01 ^d^	2.56 ± 0.03 ^e^
6	C20:3n-3	ETA	0.24 ± 0.04 ^b^	0.28 ± 0.01 ^b^	0.51 ± 0.14 ^a^	0.46 ± 0.00 ^a^	0.37 ± 0.08 ^a,b^	0.30 ± 0.01 ^b^	0.36 ± 0.02 ^a,b^	0.30 ± 0.01 ^d^	0.24 ± 0.01 ^e^	0.59 ± 0.01 ^a^	0.38 ± 0.00 ^c^	0.40 ± 0.05 ^c^	0.45 ± 0.00 ^b^	0.28 ± 0.00 ^d,e^
7	C20:4n-6	AA	9.24 ± 0.17 ^a^	7.42 ± 0.20 ^b,c^	7.61 ± 0.12 ^b^	7.11 ± 0.04 ^c^	6.20 ± 0.20 ^d^	6.03 ± 0.07 ^d^	6.22 ± 0.00 ^d^	8.89 ± 0.23 ^a^	7.29 ± 0.02 ^c^	7.85 ± 0.07 ^b^	7.66 ± 0.01 ^b^	6.96 ± 0.02 ^d^	6.10 ± 0.03 ^e^	6.86 ± 0.01 ^d^
8	C22:2n-6	DDAn-6	0.43 ± 0.04 ^b,c^	0.79 ± 0.06 ^a^	0.28 ± 0.01 ^d^	0.42 ± 0.01 ^b,c^	0.38 ± 0.01 ^c^	0.45 ± 0.01 ^b^	0.38 ± 0.01 ^c^	0.34 ± 0.01 ^c^	0.56 ± 0.08 ^a,b^	0.30 ± 0.00 ^c^	0.64 ± 0.02 ^a^	0.48 ± 0.20 ^a,b,c^	0.37 ± 0.00 ^b,c^	0.28 ± 0.00 ^c^
9	C20:5n-3	EPA	2.36 ± 0.14 ^b^	2.96 ± 0.02 ^a^	2.33 ± 0.01 ^b^	1.73 ± 0.01 ^d^	2.12 ± 0.00 ^c^	1.46 ± 0.02 ^e^	1.71 ± 0.02 ^d^	2.17 ± 0.04 ^c^	2.85 ± 0.04 ^a^	2.33 ± 0.01 ^b^	2.02 ± 0.05 ^d^	1.99 ± 0.10 ^d^	1.85 ± 0.01 ^e^	1.72 ± 0.00 ^f^
10	C22:5n-3	DPA	2.56 ± 0.01 ^b^	2.95 ± 0.03 ^a^	2.61 ± 0.09 ^b^	2.20 ± 0.04 ^c^	2.27 ± 0.01 ^c^	2.08 ± 0.02 ^d^	2.22 ± 0.05 ^c^	2.65 ± 0.10 ^a^	2.21 ± 0.03 ^c^	2.17 ± 0.08 ^c^	2.60 ± 0.01 ^a^	2.34 ± 0.01 ^b^	2.17 ± 0.02 ^c^	2.38 ± 0.00 ^b^
11	C22:6n-3	DHA	33.26 ± 0.04 ^a^	32.54 ± 0.26 ^b^	31.72 ± 0.00 ^c^	30.26 ± 0.05 ^d^	29.10 ± 0.27 ^e^	27.71 ± 0.10 ^f^	26.84 ± 0.05 ^g^	34.22 ± 0.07 ^a^	33.01 ± 0.22 ^b^	30.37 ± 0.18 ^c^	28.35 ± 0.00 ^d^	26.98 ± 0.10 ^e^	24.44 ± 0.09 ^f^	22.46 ± 0.01 ^g^
	PUFA		55.03 ± 0.07 ^a^	53.68 ± 0.50 ^b^	51.79 ± 0.11 ^c^	50.08 ± 0.11 ^d^	47.84 ± 0.25 ^e^	45.01 ± 0.05 ^f^	43.41 ± 0.18 ^g^	54.28 ± 0.11 ^a^	53.23 ± 0.12 ^b^	49.83 ± 0.21 ^c^	48.37 ± 0.09 ^d^	45.26 ± 0.13 ^e^	41.29 ± 0.18 ^f^	39.10 ± 0.07 ^g^
	SFA		31.99 ± 0.14 ^g^	34.46 ± 0.00 ^f^	35.53 ± 0.18 ^e^	37.90 ± 0.02 ^d^	41.11 ± 0.17 ^c^	45.84 ± 0.25 ^b^	49.75 ± 0.09 ^a^	31.99 ± 0.14 ^g^	34.46 ± 0.00 ^f^	35.53 ± 0.18 ^e^	37.90 ± 0.02 ^d^	41.11 ± 0.17 ^c^	45.84 ± 0.25 ^b^	49.75 ± 0.09 ^a^
	MUFA		13.73 ± 0.25 ^b^	12.31 ± 0.12 ^d^	14.64 ± 0.03 ^a^	13.73 ± 0.07 ^b^	13.63 ± 0.04 ^b^	12.87 ± 0.07 ^c^	11.15 ± 0.02 ^e^	13.73 ± 0.25 ^b^	12.31 ± 0.12 ^d^	14.64 ± 0.03 ^a^	13.73 ± 0.07 ^b^	13.63 ± 0.04 ^b^	12.87 ± 0.07 ^c^	11.15 ± 0.02 ^e^

Note: Different letters in the same row represent significant differences between the results (*p* < 0.05). LA: Linoleic acid; γ-LA: γ-Linolenic acid; ALA: α-Linolenic acid; EDA: Eicosadienoic acid; DGLA: 8,11,14-Eicosatrienoic acid; ETA: Eicosatrienoic acid; AA: Arachidonic acid; DDAn-6: Docosahrodienoic acid; EPA: Eicosapentaenoic acid; DPA: Docosapentaenoic acid; DHA: Docosahexaenoic acid; PUFA: Polyunsaturated fatty acid; SFA: Saturated fatty acid; MUFA: Monounsaturated fatty acid.

**Table 6:** Change in texture properties of blue round scad samples during storage.

Storage Temperature (°C)	Storage Time	EG				CG			
		Hardness (N)	Elasticity (mm)	Cohesion	Chewiness (mJ)	Hardness (N)	Elasticity (mm)	Cohesion	Chewiness (mJ)
4	0 d	10.92 ± 0.82 ^a^	1.31 ± 0.10 ^a^	1.05 ± 0.18 ^a^	9.64 ± 0.47 ^a^	8.79 ± 0.64 ^a^	1.45 ± 0.15 ^a^	0.79 ± 0.05 ^a^	9.22 ± 0.50 ^a^
	3 d	9.20 ± 0.28 ^b,c^	0.97 ± 0.03 ^b^	0.95 ± 0.21 ^a,b^	9.03 ± 0.57 ^a^	8.00 ± 0.19 ^a,b^	1.42 ± 0.07 ^a^	0.68 ± 0.04 ^a^	7.91 ± 1.15 ^b^
	6 d	9.44 ± 0.38 ^b^	1.16 ± 0.26 ^a,b^	0.75 ± 0.06 ^b^	7.42 ± 0.38 ^b^	7.23 ± 0.48 ^b,c^	1.32 ± 0.14 ^a^	0.51 ± 0.04 ^b^	6.37 ± 0.12 ^c^
	9 d	8.37 ± 0.43 ^c^	1.01 ± 0.18 ^a,b^	0.72 ± 0.03 ^b^	6.69 ± 0.44 ^b^	6.58 ± 0.70 ^c^	1.19 ± 0.23 ^a^	0.37 ± 0.10 ^c^	5.87 ± 0.55 ^c^
25	0 h	10.92 ± 0.82 ^a^	1.31 ± 0.10 ^a^	1.05 ± 0.18 ^a^	9.64 ± 0.47 ^a^	8.79 ± 0.64 ^a^	1.45 ± 0.15 ^a^	0.79 ± 0.05 ^a^	9.22 ± 0.50 ^a^
	12 h	8.88 ± 0.64 ^b^	1.20 ± 0.23 ^a,b^	0.99 ± 0.13 ^a,b^	9.10 ± 0.26 ^a,b^	8.03 ± 0.22 ^a^	1.40 ± 0.23 ^a^	0.67 ± 0.04 ^b^	8.33 ± 0.32 ^a,b^
	24 h	7.59 ± 0.85 ^c^	0.89 ± 0.16 ^c^	0.83 ± 0.07 ^b^	8.70 ± 0.44 ^b^	7.00 ± 0.62 ^b^	1.30 ± 0.04 ^a^	0.62 ± 0.03 ^b^	8.03 ± 0.31 ^b^
	36 h	6.74 ± 0.51 ^c^	0.97 ± 0.12 ^b,c^	0.92 ± 0.07 ^a,b^	7.55 ± 0.26 ^c^	6.42 ± 0.22 ^b^	1.21 ± 0.06 ^a,b^	0.53 ± 0.03 ^c^	6.23 ± 0.38 ^c^
	48 h	6.32 ± 0.53 ^c^	0.85 ± 0.07 ^c^	0.80 ± 0.06 ^b^	7.13 ± 0.31 ^c^	4.87 ± 0.63 ^c^	0.98 ± 0.10 ^b^	0.38 ± 0.04 ^d^	4.97 ± 0.86 ^d^
37	0 h	10.92 ± 0.82 ^a^	1.31 ± 0.10 ^a^	1.05 ± 0.18 ^a^	9.64 ± 0.47 ^a^	8.79 ± 0.64 ^a^	1.45 ± 0.15 ^a^	0.79 ± 0.05 ^a^	9.22 ± 0.50 ^a^
	1 h	9.15 ± 0.64 ^b^	1.16 ± 0.03 ^a,b^	0.92 ± 0.05 ^a,b^	9.17 ± 0.40 ^a^	7.87 ± 0.41 ^a,b^	1.23 ± 0.04 ^b^	0.66 ± 0.09 ^b^	7.33 ± 0.47 ^b,c^
	2 h	8.53 ± 0.21 ^b^	1.01 ± 0.11 ^b,c^	0.82 ± 0.02 ^b,c^	9.47 ± 0.25 ^a^	7.93 ± 0.85 ^a,b^	1.14 ± 0.08 ^b,c^	0.60 ± 0.02 ^b^	7.60 ± 1.04 ^b^
	3 h	9.69 ± 0.35 ^a,b^	1.04 ± 0.06 ^b,c^	0.95 ± 0.04 ^a,b^	8.17 ± 0.25 ^b^	6.93 ± 0.61 ^b^	0.94 ± 0.01 ^d,e^	0.50 ± 0.03 ^c^	5.93 ± 1.08 ^c,d^
	4 h	7.04 ± 1.49 ^c^	0.88 ± 0.12 ^c^	0.72 ± 0.02 ^c,d^	6.97 ± 0.49 ^c^	5.84 ± 0.57 ^c^	1.11 ± 0.07 ^b,c,d^	0.41 ± 0.05 ^c,d^	4.97 ± 1.01 ^d,e^
	5 h	6.75 ± 0.10 ^c^	0.94 ± 0.10 ^c^	0.62 ± 0.05 ^d^	5.40 ± 0.50 ^d^	5.23 ± 0.50 ^c^	0.97 ± 0.14 ^c,d,e^	0.37 ± 0.06 ^d,e^	4.23 ± 0.38 ^e^
	6 h	6.25 ± 0.93 ^c^	0.71 ± 0.04 ^d^	0.60 ± 0.07 ^d^	5.07 ± 0.25 ^d^	4.92 ± 0.56 ^c^	0.89 ± 0.09 ^e^	0.29 ± 0.05 ^e^	4.67 ± 0.90 ^d,e^

Note: Different letters in the same column at each storage temperature represent significant differences between the results (*p* < 0.05).

**Table 7:** Correlations between sensory score and changes in physical and chemical indices of blue round scad samples stored at different temperatures.

	Storage Temperature (°C)	TBARS	TVB-N	pH	Hardness (N)	Elasticity	Cohesion	Chewiness
EG	4	−0.960 *	−0.934	−0.978 *	0.928	0.654	0.963 *	0.97 *
	25	−0.951 *	−0.922 *	−0.137	0.998 *	0.936 *	0.886 *	0.944 *
	37	−0.988 **	−0.989 **	−0.799 *	0.918 **	0.925 **	0.933 **	0.967 **
CG	4	−0.966 *	−0.953 *	−0.998 **	0.993 **	0.915	0.976 *	0.996 **
	25	−0.950 *	−0.971 **	−0.756	0.964 **	0.910 *	0.954 *	0.914 *
	37	−0.996 **	−0.979 **	−0.917 **	0.962 **	0.868 *	0.968 **	0.902 **

Note: * indicates the correlation is significant (*p* < 0.05); ** indicates the very significant correlation (*p* < 0.01).

**Table 8:** Regression equations of changing TBARS value for EG samples at different storage temperatures.

Temperature (K)	Regression Equation	Regression Coefficient (R^2^)	Constant (*k*)
277.15	Y = 0.00138X + 0.084	0.94736	0.00138
298.15	Y = 0.00632X + 0.0938	0.9743	0.00632
310.15	Y = 0.06356X + 0.07782	0.97667	0.06356

**Table 9:** Regression equations for change in TBARS value for CG samples at different storage temperatures.

Temperature (K)	Regression Equation	Regression Coefficient (R^2)^	Constant (*k*)
277.15	Y = 0.00205X + 0.08364	0.98298	0.00205
298.15	Y = 0.01005X + 0.08128	0.97781	0.01005
310.15	Y = 0.07905X + 0.07322	0.98496	0.07905

**Table 10:** Comparison of predicted and actual values for the shelf life of blue round scad at different storage temperatures.

	Temperature (K)	Actual Value	Predicted Value	Relative Error (%)
EG	277.15	50 d	55 d	9%
	298.15	2.5 d	2.7 d	7.4%
	310.15	26 h	28 h	7.1%
CG	277.15	36 d	38 d	5.3%
	298.15	1.4 d	1.5 d	6.7%
	310.15	19 h	20 h	5.0%

Note: Different letters in the same column at each storage temperature represent significant differences between the results (*p* < 0.05).

## Data Availability

The original contributions presented in the study are included in the article; further inquiries can be directed to the corresponding author.
